# *N*-Benzyl-*N*-methyl-3-phenyl-3-[4-(tri­fluoro­meth­yl)phen­oxy]propanamine (*N*-benzylflouoxetine)

**DOI:** 10.1107/S1600536810012699

**Published:** 2010-04-24

**Authors:** Nosheen Kanwal, Erum Akbar Hussain, Onur Sahin

**Affiliations:** a Department of Chemistry, Lahore College for Women University, Lahore 54000, Pakistan; b Department of Physics, Ondokuz Mayıs University, TR-55139, Samsun, Turkey

## Abstract

In the title compound, C_24_H_24_F_3_NO, the *N*-benzyl derivative of fluoxetine {*N*-methyl-3-[4-(trifluoro­meth­yl)phen­oxy]­benzene­propanamine}, the three aromatic rings *A*, *B* and *C* are inclined to one another by 76.77 (12)° for *A*/*B*, 17.05 (14)° for *A*/*C* and 89.66 (14)° for *B*/*C*. In the crystal structure, mol­ecules are linked *via* C—H⋯π inter­actions to form one-dimensional chains propagating in the [010] direction.

## Related literature

For the therapeutic uses of fluoxetine, see: Benefield *et al.* (1986 ▶); Feighner & Boyer (1991 ▶); Markowitz *et al.* (1999 ▶); Wong *et al.* (1995 ▶); Zhu *et al.* (2009 ▶). For the crystal structures of various fluoxetine derivatives, see: Childs *et al.* (2004 ▶); Robertson *et al.* (1988 ▶).
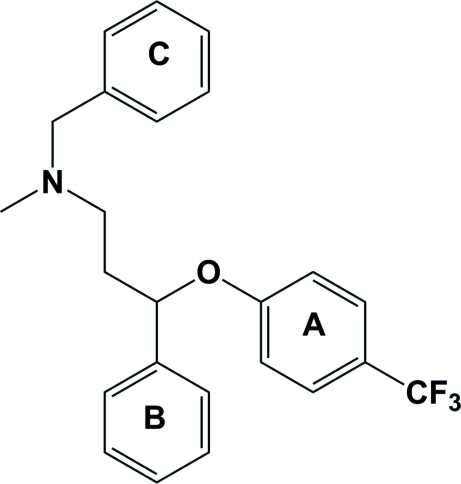


## Experimental

### 

#### Crystal data

C_24_H_24_F_3_NO
*M* *_r_* = 399.44
Monoclinic, 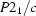

*a* = 6.1712 (5) Å
*b* = 17.2900 (14) Å
*c* = 20.3028 (16) Å
β = 91.029 (5)°
*V* = 2166.0 (3) Å^3^
*Z* = 4
Mo *K*α radiation
μ = 0.09 mm^−1^
*T* = 296 K
0.31 × 0.25 × 0.22 mm

#### Data collection

Bruker APEXII CCD area-detector diffractometer
24582 measured reflections
5395 independent reflections
1743 reflections with *I* > 2σ(*I*)
*R*_int_ = 0.092

#### Refinement

*R*[*F* ^2^ > 2σ(*F* ^2^)] = 0.052
*wR*(*F* ^2^) = 0.136
*S* = 0.91
5395 reflections
297 parameters
8 restraints
H-atom parameters constrained
Δρ_max_ = 0.13 e Å^−3^
Δρ_min_ = −0.12 e Å^−3^

### 

Data collection: *APEX2* (Bruker, 2007 ▶); cell refinement: *SAINT* (Bruker, 2007 ▶); data reduction: *SAINT*; program(s) used to solve structure: *SHELXS97* (Sheldrick, 2008 ▶); program(s) used to refine structure: *SHELXL97* (Sheldrick, 2008 ▶); molecular graphics: *ORTEP-3 for Windows* (Farrugia, 1997 ▶); software used to prepare material for publication: *WinGX* (Farrugia, 1999 ▶).

## Supplementary Material

Crystal structure: contains datablocks global, I. DOI: 10.1107/S1600536810012699/su2167sup1.cif

Structure factors: contains datablocks I. DOI: 10.1107/S1600536810012699/su2167Isup2.hkl

Additional supplementary materials:  crystallographic information; 3D view; checkCIF report

## Figures and Tables

**Table 1:** C—H⋯π inter­actions (Å, °) *Cg*1 is the centroid of ring *A* (C1–C6), *Cg*2 that of ring *B* (C8–C13) and *Cg*3 that of ring *C* (C17–C22).

*D*	H	Centroid	C—H	H⋯*Cg*	*D*⋯*Cg*	C—H⋯*Cg*
C10	H10	*Cg*3^i^	0.93	2.90	3.588 (3)	132
C18	H18	*Cg*1^ii^	0.93	3.08	3.976 (4)	162
C19	H19	*Cg*2^ii^	0.93	2.94	3.719 (4)	143

Symmetry codes: (i) −*x* + 1, *y* − 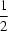, −*z* + 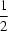; (ii) −*x*, *y* + 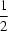, −*z* + 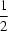.

**Fig. 1.**
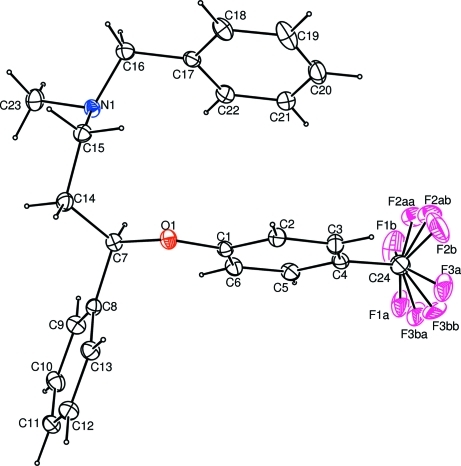
A view of the three independent molecules of the title compound, showing the atom-numbering scheme and 30% probability displacement ellipsoids.

**Fig. 2.**
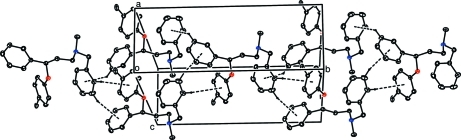
Part of the crystal packing of the title compound, showing the formation of a chain along [010], generated by the C—H···π interactions [For clarity the H and F atoms not involved in the motifs shown have been omitted].

## supplementary crystallographic information

### Comment

Fluoxetine (*N*-methyl-3-[4-(trifluoromethyl)phenoxy]benzenepropanamine) has been approved worldwide in the therapy of major depression (Markowitz *et al.*, 1999); Feighner & Boyer, 1991) and in the treatment of other syndromes, such as Bulimia nervosa, Panic fits and obsessive–compulsive disorder (Benefield *et al.*, 1986; Wong *et al.*, 1995). Recently, Zhu *et al.* reported that continuous Fluoxetine administration also prevents recurrence of pulmonary arterial hypertension in rats (Zhu *et al.*, 2009). Crystal structure of Fluoxetine has been reported as the hydrochloride, hydrochloride benzoic acid, hydrochloride succinic acid and hydrochloride fumaric acid (Robertson *et al.*, 1988; Childs *et al.*, 2004). Herein, we report on the crystal structure of *N*-Benzyl Fluoxetine.

The molecular structure of the title molecule is illustrated in Fig. 1. The geometrical parameters are similar to those in the above mentioned derivatives. In the title compound the F atoms of the CF_3_ groups shows disorder and were modelled with three different orientations (F1a—F3a, F1b—F2b and F2aa—F2ab—F3bb—F3ba) with occupancy factors of 0.50, 0.50 and 0.25, respectively (Fig. 1). The H7—C7—C8—C9 torsion angle is -19.2°, indicating that the monosubstituted phenyl ring (B) deviates only slightly from the plane defined by atoms C8, C7, and H7.

The relationship of this phenyl ring to the trifluoromethyl-substituted phenoxy ring (A) is defined by the torsion angles C8—C7—O1—C1 and C7—O1—C1—C6, which are 82.8 (2) and -6.9 (3)°, respectively. The three phenyl ring mean planes are approximately planar, with maximum deviations of 0.0094 (17) Å for atom C3 (ring A), 0.0032 (18) Å for atom C11 (ring B) and 0.0050 (17) Å for atom C17 (ring C).

In the crystal structure of the title compound, there are no intra- or intermolecular hydrogen-bonding interactions, only weak C—H···π interactions. These lead to the formation of a chain propagating along [010]; see Fig. 2 and Table 1.

### Experimental

A mixture of Fluoxetine hydrogen chloride 0.5 g (1.45 mmol), sodium hydride 0.14 g (5.8 mmol) and *N*,*N*-dimethylformamide (10 ml) was stirred at room temperature for 30 min, followed by the addition of benzyl chloride 0.33 ml (2.9 mmol). Stirring was continued for a period of 3 h and the contents were then poured over crushed ice. The precipitated product was isolated, washed and crystallized from methanol, giving colourless prism-like crystals, suitable for X-ray analysis.

### Refinement

The F atoms of the CF_3_ group shows disorder and they were modelled with three different orientations (F1a/F3a, F1b/F2b and F2aa/F2ab/F3bb/F3ba) with occupancy factors of 0.50, 0.50 and 0.25, respectively The C-bound H atoms were included in calculated positions and refined using a riding model: C—H = 0.98, 0.97, 0.96 and 0.93 Å, for methine, methylene, methyl and aromatic H atoms, respectively, with U_iso_(H) = k × U_eq_(C), where k = 1.2 for methine, methylene and aromatic H atoms and = 1.5 for methyl H atoms.

### Crystal data

**Table:**

C_24_H_24_F_3_NO	*F*(000) = 840
*M**_r_* = 399.44	*D*_x_ = 1.225 Mg m^−^^3^
Monoclinic, *P*2_1_/*c*	Mo *K*α radiation, λ = 0.71073 Å
Hall symbol: -P 2ybc	Cell parameters from 1693 reflections
*a* = 6.1712 (5) Å	θ = 3.1–17.9°
*b* = 17.2900 (14) Å	µ = 0.09 mm^−^^1^
*c* = 20.3028 (16) Å	*T* = 296 K
β = 91.029 (5)°	Prism, colourless
*V* = 2166.0 (3) Å^3^	0.31 × 0.25 × 0.22 mm
*Z* = 4	

### Data collection

**Table:**

Bruker APEXII CCD area-detector diffractometer	1743 reflections with *I* > 2σ(*I*)
Radiation source: fine-focus sealed tube	*R*_int_ = 0.092
graphite	θ_max_ = 28.4°, θ_min_ = 2.3°
φ and ω scans	*h* = −8→8
24582 measured reflections	*k* = −20→23
5395 independent reflections	*l* = −27→26

### Refinement

**Table:**

Refinement on *F*^2^	Secondary atom site location: difference Fourier map
Least-squares matrix: full	Hydrogen site location: inferred from neighbouring sites
*R*[*F*^2^ > 2σ(*F*^2^)] = 0.052	H-atom parameters constrained
*wR*(*F*^2^) = 0.136	*w* = 1/[σ^2^(*F*_o_^2^) + (0.0453*P*)^2^] where *P* = (*F*_o_^2^ + 2*F*_c_^2^)/3
*S* = 0.91	(Δ/σ)_max_ < 0.001
5395 reflections	Δρ_max_ = 0.13 e Å^−^^3^
297 parameters	Δρ_min_ = −0.12 e Å^−^^3^
8 restraints	Extinction correction: *SHELXL97* (Sheldrick, 2008), Fc^*^ = kFc[1+0.001xFc^2^λ^3^/sin(2θ)]^-1/4^
Primary atom site location: structure-invariant direct methods	Extinction coefficient: 0.0059 (9)

### Special details

**Table:**

Geometry. All esds (except the esd in the dihedral angle between two l.s. planes) are estimated using the full covariance matrix. The cell esds are taken into account individually in the estimation of esds in distances, angles and torsion angles; correlations between esds in cell parameters are only used when they are defined by crystal symmetry. An approximate (isotropic) treatment of cell esds is used for estimating esds involving l.s. planes.
Refinement. Refinement of *F*^2^ against ALL reflections. The weighted *R*-factor wR and goodness of fit *S* are based on *F*^2^, conventional *R*-factors *R* are based on *F*, with *F* set to zero for negative *F*^2^. The threshold expression of *F*^2^ > σ(*F*^2^) is used only for calculating *R*-factors(gt) etc. and is not relevant to the choice of reflections for refinement. *R*-factors based on *F*^2^ are statistically about twice as large as those based on *F*, and *R*- factors based on ALL data will be even larger.

### Fractional atomic coordinates and isotropic or equivalent isotropic displacement parameters (Å^2^)

**Table:**

	*x*	*y*	*z*	*U*_iso_*/*U*_eq_	Occ. (<1)
O1	0.0963 (2)	0.50665 (8)	0.16926 (7)	0.0583 (4)	
N1	0.5187 (3)	0.65181 (10)	0.17839 (9)	0.0558 (5)	
C1	0.0584 (4)	0.47323 (13)	0.22867 (11)	0.0502 (6)	
C2	−0.1392 (4)	0.49238 (13)	0.25629 (12)	0.0594 (6)	
H2	−0.2349	0.5248	0.2337	0.071*	
C3	−0.1931 (4)	0.46374 (15)	0.31639 (13)	0.0680 (7)	
H3	−0.3265	0.4761	0.3342	0.082*	
C4	−0.0517 (5)	0.41668 (14)	0.35088 (12)	0.0639 (7)	
C5	0.1420 (4)	0.39704 (14)	0.32336 (13)	0.0689 (7)	
H5	0.2372	0.3647	0.3462	0.083*	
C6	0.1974 (4)	0.42465 (13)	0.26226 (12)	0.0620 (7)	
H6	0.3284	0.4104	0.2438	0.074*	
C7	0.3076 (3)	0.49854 (14)	0.14017 (11)	0.0543 (6)	
H7	0.4190	0.5008	0.1751	0.065*	
C8	0.3265 (4)	0.42297 (14)	0.10444 (11)	0.0519 (6)	
C9	0.5138 (4)	0.37997 (15)	0.10818 (12)	0.0697 (7)	
H9	0.6283	0.3970	0.1348	0.084*	
C10	0.5352 (5)	0.31207 (17)	0.07326 (14)	0.0777 (8)	
H10	0.6631	0.2838	0.0764	0.093*	
C11	0.3681 (6)	0.28656 (16)	0.03409 (13)	0.0788 (8)	
H11	0.3826	0.2410	0.0103	0.095*	
C12	0.1785 (5)	0.32795 (18)	0.02973 (13)	0.0778 (8)	
H12	0.0641	0.3104	0.0033	0.093*	
C13	0.1591 (4)	0.39583 (15)	0.06486 (12)	0.0665 (7)	
H13	0.0307	0.4238	0.0618	0.080*	
C14	0.3318 (4)	0.56805 (13)	0.09553 (11)	0.0619 (7)	
H14A	0.4648	0.5627	0.0712	0.074*	
H14B	0.2122	0.5686	0.0639	0.074*	
C15	0.3365 (3)	0.64435 (13)	0.13203 (11)	0.0598 (7)	
H15A	0.3431	0.6861	0.1002	0.072*	
H15B	0.2026	0.6500	0.1559	0.072*	
C16	0.4799 (4)	0.71459 (14)	0.22471 (12)	0.0675 (7)	
H16A	0.4284	0.7595	0.2005	0.081*	
H16B	0.6159	0.7284	0.2463	0.081*	
C17	0.3187 (4)	0.69440 (15)	0.27594 (12)	0.0594 (7)	
C18	0.1365 (5)	0.73751 (17)	0.28500 (13)	0.0794 (8)	
H18	0.1099	0.7799	0.2578	0.095*	
C19	−0.0087 (5)	0.7199 (2)	0.33326 (18)	0.1073 (12)	
H19	−0.1322	0.7500	0.3384	0.129*	
C20	0.0286 (6)	0.6584 (3)	0.37342 (17)	0.1050 (12)	
H20	−0.0696	0.6464	0.4061	0.126*	
C21	0.2097 (7)	0.61415 (18)	0.36606 (16)	0.1025 (11)	
H21	0.2351	0.5719	0.3935	0.123*	
C22	0.3546 (5)	0.63265 (16)	0.31761 (15)	0.0826 (8)	
H22	0.4789	0.6029	0.3130	0.099*	
C23	0.7204 (3)	0.66501 (15)	0.14376 (13)	0.0821 (8)	
H23A	0.7484	0.6219	0.1153	0.123*	
H23B	0.8375	0.6704	0.1752	0.123*	
H23C	0.7080	0.7114	0.1180	0.123*	
C24	−0.1069 (8)	0.3881 (3)	0.41791 (18)	0.0918 (10)	
F1A	−0.023 (2)	0.3208 (7)	0.4307 (7)	0.117 (2)	0.50
F2AA	−0.024 (5)	0.4318 (16)	0.4644 (11)	0.119 (9)	0.25
F2AB	−0.145 (4)	0.4449 (16)	0.4585 (14)	0.119 (9)	0.25
F3A	−0.3270 (11)	0.3735 (7)	0.4202 (5)	0.117 (2)	0.50
F1B	0.0799 (13)	0.3802 (13)	0.4568 (5)	0.195 (4)	0.50
F2B	−0.207 (3)	0.4342 (8)	0.4524 (7)	0.198 (8)	0.50
F3BA	−0.115 (3)	0.3139 (9)	0.4299 (11)	0.115 (7)	0.25
F3BB	−0.266 (4)	0.3368 (8)	0.4193 (7)	0.102 (4)	0.25

### Atomic displacement parameters (Å^2^)

**Table:**

	*U*^11^	*U*^22^	*U*^33^	*U*^12^	*U*^13^	*U*^23^
O1	0.0573 (10)	0.0676 (11)	0.0499 (10)	0.0091 (8)	0.0015 (8)	0.0066 (9)
N1	0.0472 (11)	0.0650 (14)	0.0553 (13)	0.0002 (10)	0.0054 (10)	−0.0031 (11)
C1	0.0585 (15)	0.0490 (16)	0.0428 (15)	−0.0006 (12)	−0.0029 (12)	−0.0009 (13)
C2	0.0586 (15)	0.0634 (17)	0.0559 (17)	0.0019 (13)	−0.0014 (13)	0.0045 (14)
C3	0.0693 (17)	0.0713 (19)	0.0638 (19)	0.0038 (14)	0.0113 (15)	0.0053 (16)
C4	0.090 (2)	0.0531 (18)	0.0488 (17)	−0.0010 (15)	0.0071 (15)	−0.0007 (14)
C5	0.093 (2)	0.0546 (17)	0.0588 (19)	0.0143 (14)	−0.0046 (16)	0.0058 (14)
C6	0.0672 (16)	0.0603 (18)	0.0586 (18)	0.0161 (13)	0.0030 (14)	0.0035 (14)
C7	0.0488 (14)	0.0631 (17)	0.0510 (15)	0.0025 (12)	−0.0011 (11)	−0.0015 (14)
C8	0.0538 (15)	0.0548 (17)	0.0472 (15)	−0.0013 (13)	0.0027 (12)	0.0036 (13)
C9	0.0674 (17)	0.071 (2)	0.0704 (19)	0.0088 (14)	−0.0047 (14)	−0.0046 (16)
C10	0.090 (2)	0.075 (2)	0.068 (2)	0.0236 (17)	0.0081 (16)	−0.0004 (17)
C11	0.115 (2)	0.065 (2)	0.0565 (19)	−0.0038 (19)	0.0126 (18)	−0.0072 (15)
C12	0.088 (2)	0.082 (2)	0.0635 (19)	−0.0147 (17)	−0.0034 (15)	−0.0090 (17)
C13	0.0658 (16)	0.071 (2)	0.0626 (18)	0.0000 (14)	−0.0019 (14)	−0.0063 (15)
C14	0.0676 (16)	0.0649 (18)	0.0533 (16)	−0.0013 (13)	0.0024 (12)	0.0065 (15)
C15	0.0595 (15)	0.0558 (17)	0.0643 (17)	0.0046 (12)	0.0016 (13)	0.0036 (14)
C16	0.0691 (16)	0.0635 (18)	0.0700 (19)	−0.0056 (13)	0.0023 (15)	−0.0066 (15)
C17	0.0615 (16)	0.0561 (18)	0.0605 (18)	−0.0021 (14)	0.0034 (14)	−0.0140 (15)
C18	0.0753 (19)	0.104 (2)	0.0590 (19)	0.0177 (18)	−0.0067 (16)	−0.0164 (17)
C19	0.081 (2)	0.171 (4)	0.070 (3)	0.021 (2)	0.004 (2)	−0.037 (2)
C20	0.101 (3)	0.141 (4)	0.074 (3)	−0.037 (2)	0.028 (2)	−0.037 (3)
C21	0.150 (3)	0.074 (2)	0.086 (3)	−0.017 (2)	0.040 (2)	−0.0079 (18)
C22	0.099 (2)	0.065 (2)	0.084 (2)	0.0102 (16)	0.0231 (19)	−0.0049 (18)
C23	0.0596 (16)	0.099 (2)	0.088 (2)	−0.0046 (15)	0.0151 (15)	0.0027 (17)
C24	0.125 (4)	0.070 (3)	0.081 (3)	0.005 (3)	0.016 (3)	0.010 (3)
F1A	0.135 (4)	0.110 (4)	0.109 (3)	0.023 (3)	0.047 (3)	0.057 (3)
F2AA	0.21 (3)	0.108 (11)	0.041 (5)	−0.056 (15)	−0.015 (13)	−0.008 (7)
F2AB	0.21 (3)	0.108 (11)	0.041 (5)	−0.056 (15)	−0.015 (13)	−0.008 (7)
F3A	0.135 (4)	0.110 (4)	0.109 (3)	0.023 (3)	0.047 (3)	0.057 (3)
F1B	0.189 (7)	0.299 (13)	0.097 (5)	0.017 (9)	−0.009 (4)	0.088 (7)
F2B	0.348 (18)	0.139 (13)	0.111 (9)	0.068 (12)	0.118 (10)	0.014 (7)
F3BA	0.146 (18)	0.068 (8)	0.132 (9)	0.016 (9)	0.053 (13)	0.049 (7)
F3BB	0.158 (12)	0.032 (6)	0.116 (7)	−0.018 (7)	0.009 (8)	0.032 (6)

### Geometric parameters (Å, °)

**Table:**

O1—C1	1.361 (2)	C14—H14B	0.9700
O1—C7	1.448 (2)	C15—H15A	0.9700
N1—C23	1.458 (2)	C15—H15B	0.9700
N1—C16	1.459 (3)	C16—C17	1.493 (3)
N1—C15	1.459 (2)	C16—H16A	0.9700
C1—C6	1.373 (3)	C16—H16B	0.9700
C1—C2	1.391 (3)	C17—C18	1.364 (3)
C2—C3	1.363 (3)	C17—C22	1.378 (3)
C2—H2	0.9300	C18—C19	1.374 (4)
C3—C4	1.375 (3)	C18—H18	0.9300
C3—H3	0.9300	C19—C20	1.357 (4)
C4—C5	1.371 (3)	C19—H19	0.9300
C4—C24	1.493 (4)	C20—C21	1.365 (4)
C5—C6	1.378 (3)	C20—H20	0.9300
C5—H5	0.9300	C21—C22	1.379 (4)
C6—H6	0.9300	C21—H21	0.9300
C7—C8	1.500 (3)	C22—H22	0.9300
C7—C14	1.514 (3)	C23—H23A	0.9600
C7—H7	0.9800	C23—H23B	0.9600
C8—C9	1.375 (3)	C23—H23C	0.9600
C8—C13	1.379 (3)	C24—F2B	1.234 (10)
C9—C10	1.379 (3)	C24—F1A	1.298 (10)
C9—H9	0.9300	C24—F2AA	1.305 (15)
C10—C11	1.364 (3)	C24—F2AB	1.306 (18)
C10—H10	0.9300	C24—F3BA	1.307 (16)
C11—C12	1.373 (3)	C24—F3BB	1.323 (13)
C11—H11	0.9300	C24—F3A	1.384 (8)
C12—C13	1.380 (3)	C24—F1B	1.392 (8)
C12—H12	0.9300	F2AA—F2AB	0.79 (4)
C13—H13	0.9300	F1B—F3BA	1.74 (2)
C14—C15	1.513 (3)	F3BA—F3BB	1.03 (2)
C14—H14A	0.9700		
			
C1—O1—C7	119.38 (16)	C18—C17—C22	117.7 (3)
C23—N1—C16	110.24 (18)	C18—C17—C16	121.8 (3)
C23—N1—C15	110.93 (18)	C22—C17—C16	120.5 (2)
C16—N1—C15	110.37 (18)	C17—C18—C19	121.7 (3)
O1—C1—C6	125.7 (2)	C17—C18—H18	119.2
O1—C1—C2	115.0 (2)	C19—C18—H18	119.2
C6—C1—C2	119.3 (2)	C20—C19—C18	119.7 (3)
C3—C2—C1	120.2 (2)	C20—C19—H19	120.1
C3—C2—H2	119.9	C18—C19—H19	120.1
C1—C2—H2	119.9	C19—C20—C21	120.3 (3)
C2—C3—C4	120.5 (2)	C19—C20—H20	119.9
C2—C3—H3	119.7	C21—C20—H20	119.9
C4—C3—H3	119.7	C20—C21—C22	119.4 (3)
C5—C4—C3	119.2 (2)	C20—C21—H21	120.3
C5—C4—C24	120.3 (3)	C22—C21—H21	120.3
C3—C4—C24	120.5 (3)	C17—C22—C21	121.3 (3)
C4—C5—C6	120.9 (2)	C17—C22—H22	119.4
C4—C5—H5	119.6	C21—C22—H22	119.4
C6—C5—H5	119.6	N1—C23—H23A	109.5
C1—C6—C5	119.8 (2)	N1—C23—H23B	109.5
C1—C6—H6	120.1	H23A—C23—H23B	109.5
C5—C6—H6	120.1	N1—C23—H23C	109.5
O1—C7—C8	111.09 (17)	H23A—C23—H23C	109.5
O1—C7—C14	105.45 (17)	H23B—C23—H23C	109.5
C8—C7—C14	113.11 (19)	F2B—C24—F1A	131.9 (9)
O1—C7—H7	109.0	F2B—C24—F2AA	53.7 (12)
C8—C7—H7	109.0	F1A—C24—F2AA	103.0 (14)
C14—C7—H7	109.0	F1A—C24—F2AB	128.6 (15)
C9—C8—C13	117.9 (2)	F2B—C24—F3BA	120.5 (10)
C9—C8—C7	121.1 (2)	F2AA—C24—F3BA	116.7 (17)
C13—C8—C7	121.0 (2)	F2AB—C24—F3BA	128 (2)
C8—C9—C10	121.3 (2)	F2B—C24—F3BB	92.5 (11)
C8—C9—H9	119.3	F1A—C24—F3BB	71.8 (8)
C10—C9—H9	119.3	F2AA—C24—F3BB	130.8 (15)
C11—C10—C9	119.8 (3)	F2AB—C24—F3BB	110.5 (17)
C11—C10—H10	120.1	F3BA—C24—F3BB	46.2 (9)
C9—C10—H10	120.1	F2B—C24—F3A	66.2 (9)
C10—C11—C12	120.2 (3)	F1A—C24—F3A	102.7 (6)
C10—C11—H11	119.9	F2AA—C24—F3A	116.9 (13)
C12—C11—H11	119.9	F2AB—C24—F3A	85.9 (13)
C11—C12—C13	119.5 (2)	F3BA—C24—F3A	77.0 (8)
C11—C12—H12	120.3	F2B—C24—F1B	99.1 (9)
C13—C12—H12	120.3	F1A—C24—F1B	58.3 (7)
C8—C13—C12	121.3 (2)	F2AA—C24—F1B	48.6 (9)
C8—C13—H13	119.3	F2AB—C24—F1B	82.7 (12)
C12—C13—H13	119.3	F3BA—C24—F1B	80.3 (11)
C15—C14—C7	113.6 (2)	F3BB—C24—F1B	121.9 (8)
C15—C14—H14A	108.8	F3A—C24—F1B	140.1 (5)
C7—C14—H14A	108.8	F2B—C24—C4	115.4 (8)
C15—C14—H14B	108.8	F1A—C24—C4	112.4 (6)
C7—C14—H14B	108.8	F2AA—C24—C4	112.0 (14)
H14A—C14—H14B	107.7	F2AB—C24—C4	111.9 (17)
N1—C15—C14	113.64 (18)	F3BA—C24—C4	120.3 (10)
N1—C15—H15A	108.8	F3BB—C24—C4	115.0 (7)
C14—C15—H15A	108.8	F3A—C24—C4	109.3 (5)
N1—C15—H15B	108.8	F1B—C24—C4	110.4 (4)
C14—C15—H15B	108.8	F2AB—F2AA—C24	72.6 (18)
H15A—C15—H15B	107.7	F2AA—F2AB—C24	72 (2)
N1—C16—C17	113.22 (18)	C24—F1B—F3BA	47.7 (7)
N1—C16—H16A	108.9	F3BB—F3BA—C24	67.7 (10)
C17—C16—H16A	108.9	F3BB—F3BA—F1B	115.4 (15)
N1—C16—H16B	108.9	C24—F3BA—F1B	52.0 (7)
C17—C16—H16B	108.9	F3BA—F3BB—C24	66.1 (12)
H16A—C16—H16B	107.7		
			
C7—O1—C1—C6	−6.9 (3)	C3—C4—C24—F3BA	122.0 (11)
C7—O1—C1—C2	172.21 (18)	C5—C4—C24—F3BB	−111.0 (12)
O1—C1—C2—C3	−178.6 (2)	C3—C4—C24—F3BB	69.8 (12)
C6—C1—C2—C3	0.6 (3)	C5—C4—C24—F3A	−144.7 (7)
C1—C2—C3—C4	1.1 (4)	C3—C4—C24—F3A	36.1 (8)
C2—C3—C4—C5	−1.8 (4)	C5—C4—C24—F1B	31.7 (12)
C2—C3—C4—C24	177.4 (3)	C3—C4—C24—F1B	−147.5 (11)
C3—C4—C5—C6	0.9 (4)	F2B—C24—F2AA—F2AB	−9(4)
C24—C4—C5—C6	−178.3 (3)	F1A—C24—F2AA—F2AB	−142 (4)
O1—C1—C6—C5	177.6 (2)	F3BA—C24—F2AA—F2AB	−119 (4)
C2—C1—C6—C5	−1.5 (3)	F3BB—C24—F2AA—F2AB	−65 (5)
C4—C5—C6—C1	0.8 (4)	F3A—C24—F2AA—F2AB	−30 (5)
C1—O1—C7—C8	82.8 (2)	F1B—C24—F2AA—F2AB	−165 (5)
C1—O1—C7—C14	−154.29 (17)	C4—C24—F2AA—F2AB	97 (4)
O1—C7—C8—C9	−139.3 (2)	F2B—C24—F2AB—F2AA	158 (9)
C14—C7—C8—C9	102.3 (2)	F1A—C24—F2AB—F2AA	50 (5)
O1—C7—C8—C13	43.0 (3)	F3BA—C24—F2AB—F2AA	83 (5)
C14—C7—C8—C13	−75.3 (3)	F3BB—C24—F2AB—F2AA	133 (4)
C13—C8—C9—C10	0.4 (4)	F3A—C24—F2AB—F2AA	153 (4)
C7—C8—C9—C10	−177.3 (2)	F1B—C24—F2AB—F2AA	12 (4)
C8—C9—C10—C11	0.0 (4)	C4—C24—F2AB—F2AA	−98 (4)
C9—C10—C11—C12	−0.5 (4)	F2B—C24—F1B—F3BA	119.6 (11)
C10—C11—C12—C13	0.5 (4)	F1A—C24—F1B—F3BA	−14.4 (13)
C9—C8—C13—C12	−0.4 (4)	F2AA—C24—F1B—F3BA	139.4 (18)
C7—C8—C13—C12	177.3 (2)	F2AB—C24—F1B—F3BA	131 (2)
C11—C12—C13—C8	−0.1 (4)	F3BB—C24—F1B—F3BA	20.9 (13)
O1—C7—C14—C15	64.4 (2)	F3A—C24—F1B—F3BA	55.9 (16)
C8—C7—C14—C15	−173.96 (18)	C4—C24—F1B—F3BA	−118.8 (9)
C23—N1—C15—C14	74.1 (2)	F2B—C24—F3BA—F3BB	60 (2)
C16—N1—C15—C14	−163.38 (18)	F1A—C24—F3BA—F3BB	−176 (4)
C7—C14—C15—N1	62.1 (2)	F2AA—C24—F3BA—F3BB	122 (2)
C23—N1—C16—C17	−162.2 (2)	F2AB—C24—F3BA—F3BB	82 (2)
C15—N1—C16—C17	74.9 (2)	F3A—C24—F3BA—F3BB	8.2 (14)
N1—C16—C17—C18	−123.5 (2)	F1B—C24—F3BA—F3BB	155.2 (16)
N1—C16—C17—C22	59.1 (3)	C4—C24—F3BA—F3BB	−96.7 (13)
C22—C17—C18—C19	−0.8 (4)	F2B—C24—F3BA—F1B	−95.0 (12)
C16—C17—C18—C19	−178.3 (2)	F1A—C24—F3BA—F1B	29 (3)
C17—C18—C19—C20	0.2 (4)	F2AA—C24—F3BA—F1B	−33.1 (12)
C18—C19—C20—C21	0.1 (5)	F2AB—C24—F3BA—F1B	−72.8 (16)
C19—C20—C21—C22	0.2 (5)	F3BB—C24—F3BA—F1B	−155.2 (16)
C18—C17—C22—C21	1.1 (4)	F3A—C24—F3BA—F1B	−146.9 (7)
C16—C17—C22—C21	178.7 (2)	C4—C24—F3BA—F1B	108.1 (8)
C20—C21—C22—C17	−0.8 (4)	C24—F1B—F3BA—F3BB	−25.5 (15)
C5—C4—C24—F2B	143.1 (14)	F1B—F3BA—F3BB—C24	21.5 (11)
C3—C4—C24—F2B	−36.1 (15)	F2B—C24—F3BB—F3BA	−131.5 (18)
C5—C4—C24—F1A	−31.4 (10)	F1A—C24—F3BB—F3BA	2(2)
C3—C4—C24—F1A	149.4 (9)	F2AA—C24—F3BB—F3BA	−90 (2)
C5—C4—C24—F2AA	84.1 (17)	F2AB—C24—F3BB—F3BA	−123 (2)
C3—C4—C24—F2AA	−95.1 (17)	F3A—C24—F3BB—F3BA	−165 (3)
C5—C4—C24—F2AB	121.9 (13)	F1B—C24—F3BB—F3BA	−29 (2)
C3—C4—C24—F2AB	−57.3 (14)	C4—C24—F3BB—F3BA	108.9 (16)
C5—C4—C24—F3BA	−58.8 (12)		

### Table 1 C—H···π interactions (Å, °)

**Table:**

D	H	Centroid	C-H	H···Cg	D···Cg	C-H···Cg
C10	H10	Cg3^i^	0.93	2.90	3.588 (3)	132
C18	H18	Cg1^ii^	0.93	3.08	3.976 (4)	162
C19	H19	Cg2^ii^	0.93	2.94	3.719 (4)	143

Cg1 is the centroid of ring A (C1–C6), Cg2 that of ring B (C8–C13) and Cg3 that of ring C (C17–C22). Symmetry codes: (i) -x + 1, y - 1/2, -z + 1/2; (ii) -x, y + 1/2, -z + 1/2.
